# No bidirectional relationship between inflammatory bowel disease and diverticular disease: a genetic correlation and Mendelian randomization study

**DOI:** 10.3389/fgene.2024.1334473

**Published:** 2024-02-14

**Authors:** Ailikamu Aierken, Falide Atabieke, Munire Aierken, Jian Li, Yu Xia, Yierzhati Aizezi, Shui-Xue Li

**Affiliations:** ^1^ Graduate School of Xinjiang Medical University, Xinjiang Uygur Autonomous Region, Urumqi, China; ^2^ The Second Department of Gastroenterology, The First Affiliated Hospital of Xinjiang Medical University, Urumqi, China; ^3^ Department of Disinfection and Vector-Borne Pathogen Control, Urumqi City Center for Disease Prevention and Control, Urumqi, China; ^4^ Center of Critical Care Medicine, First Affiliated Hospital of Xinjiang Medical University, Urumqi, China; ^5^ Department of General Surgery, Children’s Hospital of Xinjiang Uygur Autonomous Region, Urumqi, China

**Keywords:** inflammatory bowel disease, diverticular disease, Mendelian randomization, causality, Crohn’s disease, ulcerative colitis

## Abstract

**Background:** Although previous studies found that inflammatory bowel disease (IBD) and diverticular disease (DD) usually co-exist clinically, studies examining the relationship are spare.

**Aim:** Our study aspires to investigate the causal correlation between the IBD [including ulcerative colitis (UC) and Crohn’s disease (CD)] and DD using the Mendelian randomization (MR) analysis.

**Methods:** We conducted a two-sample bidirectional MR analysis using publicly available genome-wide association studies (GWAS) summary data. The single nucleotide polymorphism (SNP) data associated with DD and IBD were obtained from the Finnish Biobank and UK Biobank, respectively. Through secondary data analysis of all GWAS summary data, we systematically screened genetic instrumental variables. To address the impact of horizontal pleiotropy, several methods were employed, including the inverse variance-weighted method (IVW), maximum likelihood method, Egger regression method, weighted median method, and simple median method. These approaches aimed to detect and correct for the potential bias caused by horizontal pleiotropy.

**Results:** Genetically predicted DD did not have a causal effect on IBD (OR 1.06, 95% CI 0.98–1.17, *p* = 0.15), and had no causal effect on UC (OR 1.10, 95% CI 0.94–1.20, *p* = 0.36) and CD (OR 1.03, 95% CI 0.92–1.16, *p* = 0.62) either. Furthermore, in the reverse MR analysis, we did not observe any significant causal effect of IBD on DD. Results of complementary methods showed consistent results with those of the IVW method.

**Conclusion:** This study’s findings do not provide evidence for a causal relationship between IBD and DD, which contradicts the majority of observational studies.

## Introduction

Inflammatory bowel disease (IBD), a term frequently employed in the academic and medical fields to delineate a constellation of disorders hallmarked by enduring inflammatory processes within the gastrointestinal tract (Rahier et al., 2014), is primarily divided into two types: Crohn’s disease (CD) and ulcerative colitis (UC). IBD is more prevalent in developed or Westernized regions, with a projected global incidence of 1% by 2030 (Kaplan and Windsor, 2021; Kucharzik et al., 2021). Scholars believe that the occurrence of IBD is not coincidental but rather a result of the interplay between various factors such as genetics, immunity, and the environment (Khor et al., 2011). Under normal circumstances, the human immune system demonstrates a complex balance—being capable of recognizing and combating invading pathogens while maintaining tolerance towards host cells and symbiotic microorganisms (Kelsen and Sullivan, 2017). However, hyperreactivity or excessive responses to these symbiotic microorganisms can lead to chronic and detrimental inflammatory cascades (Cohen-Mekelburg and Waljee, 2021). In the case of IBD, it is believed that this delicate balance has been disrupted.

Diverticular disease (DD) is a common condition characterized by the presence of small pouches (diverticula) that protrude from the inner wall of the digestive tract, especially the intestines (Strate et al., 2012). It primarily affects elderly individuals in developed countries (Delvaux, 2003). These diverticula often remain asymptomatic and are typically discovered incidentally during routine colonoscopy. However, some patients may experience mild spasms, bloating, or constipation (Kurti et al., 2022). If one of these diverticula becomes inflamed or infected, it can cause severe abdominal pain, fever, nausea, and significant changes in bowel habits (Edwards, 1954). This condition is known as diverticulitis and can lead to serious complications that often require treatment, including antibiotics and pain medication. In severe cases, surgical intervention may be necessary (Denicu et al., 2022). In recent years, there has been increasing interest in the correlation, particularly the causal relationship, between IBD and DD.

There is evidence suggesting a significantly lower incidence of DD in patients with IBD (Rispo et al., 2007). However, a research endeavor undertaken by Kinnucan (Kinnucan et al., 2015) suggests that the probability of manifesting DD does not diminish in patients suffering from IBD. The intricate linkage between DD and IBD remains complex and not fully understood. Furthermore, it is imperative to recognize that all extant evidence pertaining to the correlation between IBD and DD is derived from observational studies. These studies might be subjected to potential confounding variables and could also encompass the concept of reverse causality. Therefore, there is an urgent need to develop randomized research protocols (Dong et al., 2023).

Mendelian randomization (MR) represents a burgeoning methodology that capitalizes on genetic variants, which are arbitrarily allocated at the moment of conception, as instrumental variables (IVs). This approach facilitates the deduction of causal associations between exposures and resultant outcomes (Richmond and Davey Smith, 2022). This random allocation bears resemblance to naturally occurring randomized controlled trials, assisting in offsetting the confounding impacts and reverse causation problems frequently encountered in observational investigations (Sekula et al., 2016). Therefore, we conducted an MR investigation using single nucleotide polymorphisms (SNPs) as IVs to ascertain the causal relationship between IBD and DD.

## Materials and methods

### Data sources

The collective data for DD were derived from the Finnish Biobank, capturing a comprehensive sample size of 332,580 individuals of European lineage. This sample comprised of 30,649 cases juxtaposed with 301,931 controls. The data for IBD (including UC and CD) were sourced from the IEU Open GWAS platform (https://gwas.mrcieu.ac.uk/), which is an open platform for genome-wide association studies (GWAS). The detailed information on the dataset is presented in Table 1. It is worth noting that there is no population overlap between the exposures and outcomes in the genome-wide association studies for the exposure and outcome.

**TABLE 1:** GWAS data summary information in this study.

Trait	Sample size	Number of SNPs	Populations
Inflammatory bowel disease	34,652	12,716,084	European
Ulcerative colitis	27,432	12,255,197	European
Crohn’s disease	20,883	12,276,506	European
Diverticular disease	332,580	20,169,230	European

### Study design

**FIGURE 1:**
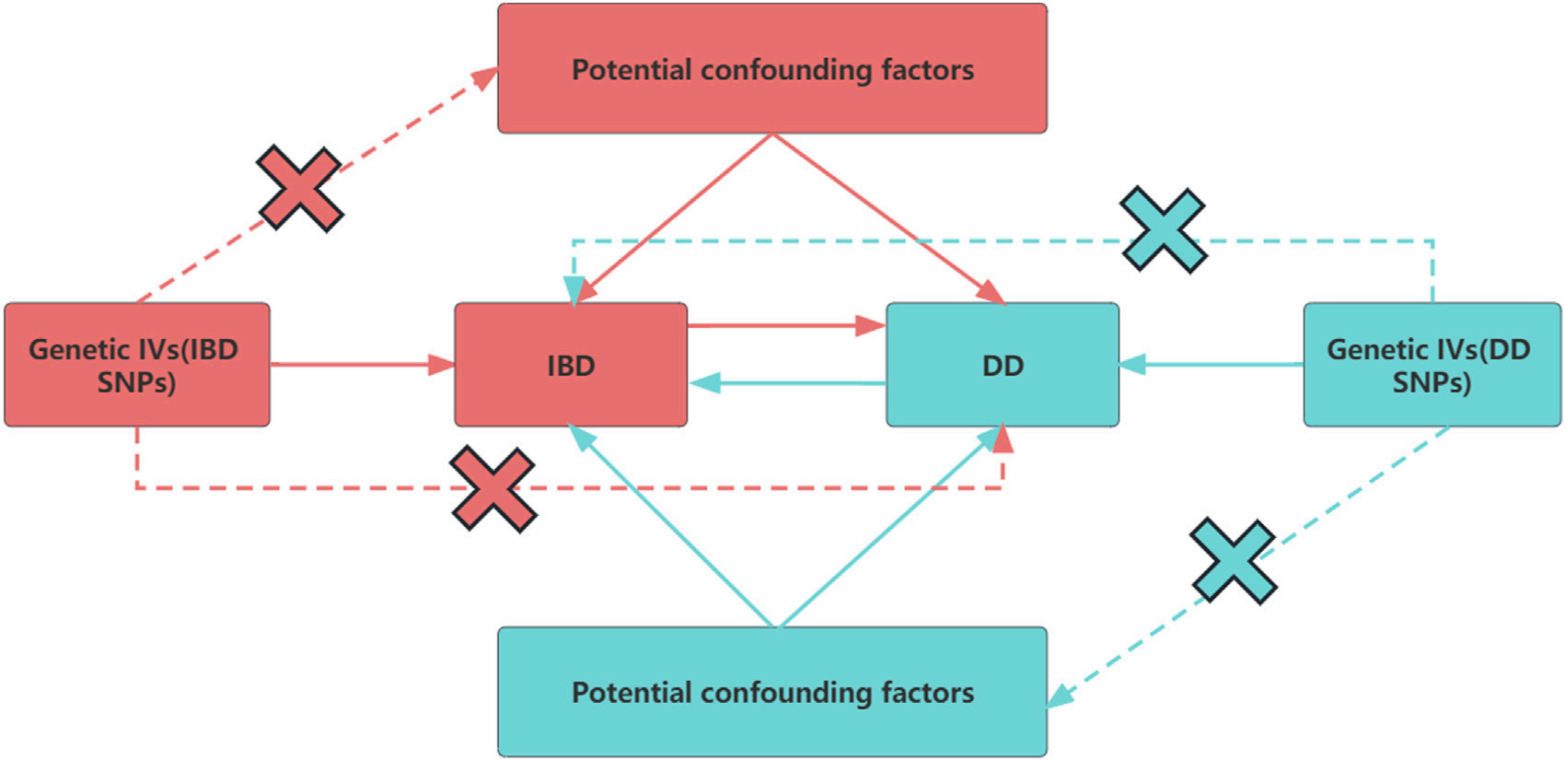
Conceptual framework diagram for Mendelian randomization analysis. IVs, instrument variants; SNP, single-nucleotide polymorphisms; IBD, inflammatory bowel disease; DD, diverticular disease.

A two-sample bidirectional MR investigation was carried out in this study, utilizing publicly accessible datasets procured from the FinnGen and the GWAS Statistics Repository. Prior to the study, informed consent was secured, and ethical approval was granted for the primary publications as well as for the utilization of these publicly accessible databases. In our MR analysis, we focused on three key assumptions, as illustrated in Figure 1: (1) the instrumental variables necessitate a robust association with DD, (2) these instrumental variables ought not to have an association with any confounding elements, and (3) the instrumental variables are permitted to exert influence on IBD exclusively through their impact on DD (Burgess et al., 2015; Evans and Davey Smith, 2015).

### Selection of tool variables

Firstly, SNPs were discerned at a threshold level of genome-wide significance (*p* < 5 × 10^−8^). Secondly, to obtain independent SNPs, SNPs were gathered based on the reference data of the European population derived from the Human Genome Project (Genomes Project Consortium et al., 2010), under the condition of linkage disequilibrium (LD) with an *r* ^2^ threshold of *r* ^2^ < 0.001 and kb > 10,000. Thirdly, we calculated the F-statistic to assess the strength of each instrumental variable, using the formula: F = *R* ^2^ × (N-2)/(1-R^2^) (Burgess et al., 2011), where N represents the sample size of DD and *R* ^2^ represents the proportion of DD variation explained by each selected SNP (Shim et al., 2015; Chen et al., 2022a). To reduce instrumental variable bias, we chose SNPs that had an F-statistic value exceeding 10. Fourthly, we utilized PhenoScanner V2 to scan for SNPs as a measure to assess the existence of confounding factors. Fifthly, genetic instrumental variables with a minimum minor allele frequency (MAF) > 0.01 were included, while excluding those located in palindrome sequences. Lastly, to meet the exclusivity assumption, we excluded SNPs directly associated with the outcome at a significance level of *p* < 5 × 10^−6^.

$$F-\text{Score}=\frac{R^{2}\left(n-k-1\right)}{n\left(1-R^{2}\right)}\times 100\%$$

$$R^{2}={\left(\frac{\beta }{se\left(\beta \right)\times \sqrt{n}}\right)}^{2}$$

### Statistical analyses

We performed analyses using five different methods, namely, the inverse variance-weighted method (IVW), maximum likelihood method, Egger regression method, weighted median method, and simple median method. Subsequently, we visualized the odds ratios (OR) and 95% confidence intervals (CI) for each of these five methods.

### Sensitivity analysis

We conducted a Cochran’s Q test to assess heterogeneity. If the resulting *p*-value is less than 0.05, it suggests the presence of heterogeneity. Subsequently, we employed the MR-Egger intercept test to evaluate horizontal pleiotropy. This test involved examining whether there is a deviation from zero in the estimated intercept. A deviation would indicate the presence of horizontal pleiotropy. Additionally, we performed a sensitivity analysis using the leave-one-out (LOO) approach to infer the presence of outliers. We also assessed the stability by observing the funnel plot results. Furthermore, we utilized the MR-PRESSO method to identify outliers and evaluate their impact on the results. All MR analyses were conducted using the TwoSampleMR package (version 0.5.6) in the R software environment (version 4.2.1).

## Results

### The causal relationship between DD and IBD

**FIGURE 2:**
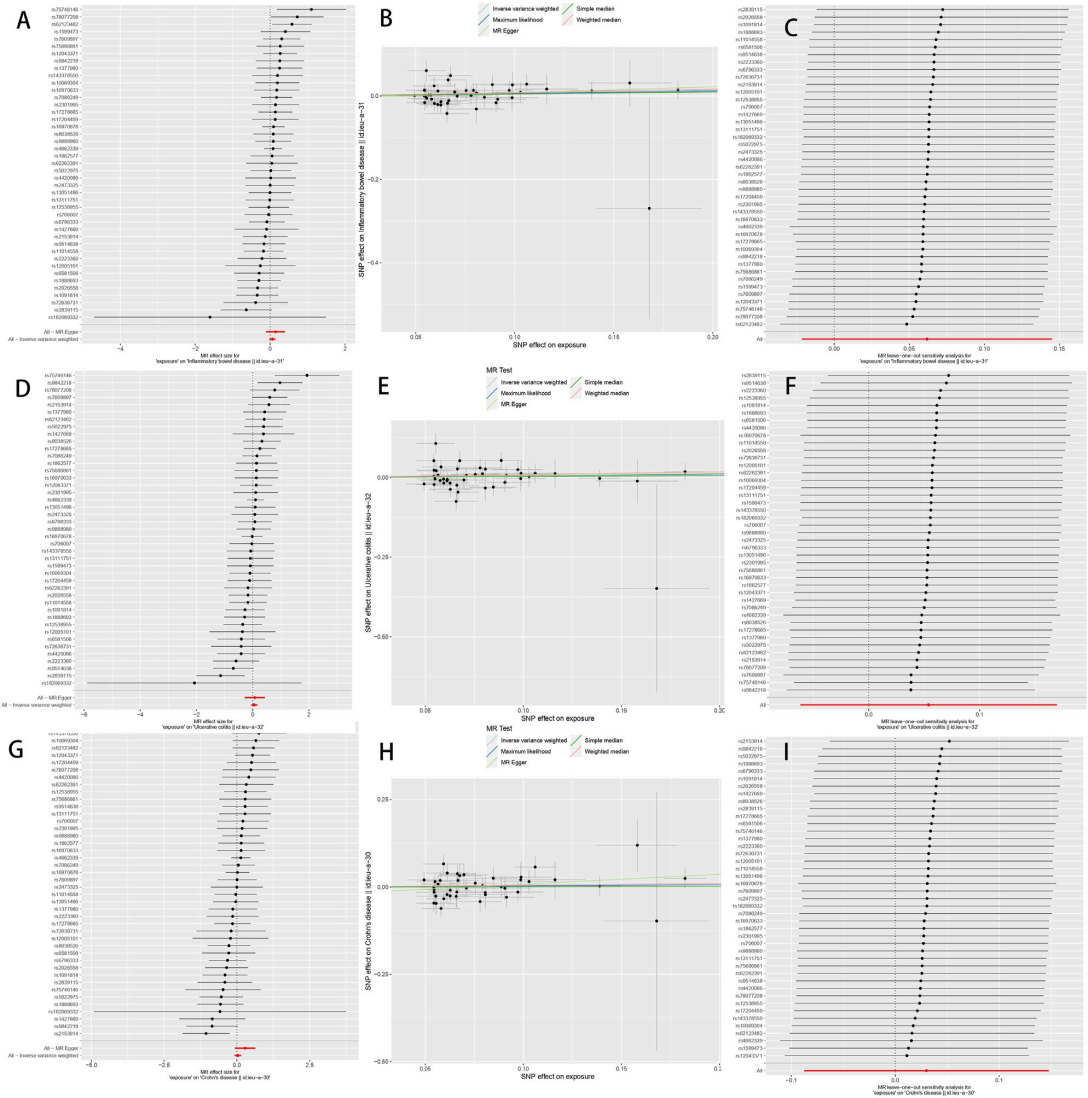
The Mendelian randomization estimate plot depicts the causal relationship between genetic predisposition to diverticular disease and the risk of inflammatory bowel disease (IBD), ulcerative colitis (UC), and Crohn’s disease (CD). **(A)** Forest plot of SNP as well as IBD risk associated with diverticular disease; **(B)** Scatter plot of SNP as well as IBD risk associated with diverticular disease; **(C)** Leave-one-out plot of SNP as well as IBD risk associated with diverticular disease; **(D)** Forest plot of SNP as well as UC risk associated with diverticular disease; **(E)** Scatter plot of SNP as well as UC risk associated with diverticular disease; **(F)** Diverticular disease associated SNPs as well as leave-one-out plots of UC risk; **(G)** Forest plots of SNPs associated with diverticular disease as well as CD risk; **(H)** Scatter plots of SNPs associated with diverticular disease as well as CD risk; and **(I)** Leave-one-out plots of SNPs associated with diverticular disease as well as CD risk. SNPs: single nucleotide polymorphisms.

Following the same selection criteria, we initially conducted a test for linkage disequilibrium to filter out SNPs that are in linkage with both DD and IBD (including UC and CD), and removed SNPs with an F-statistic below 10. We eventually identified a total of 43 SNPs associated with both DD and IBD, UC, and CD (Supplementary Table S1), of the 43 SNPs screened with MR-PRESSO and PhenoScanner, none of them need to be deleted. After implementing the threshold of *p* < 5 × 10^−6^ to select instrumental SNPs associated with the outcome, no SNPs were removed from our analysis. When DD was considered as the exposure, using the IVW random-effects model as an example, there was no significant association between DD and IBD (OR 1.06, 95% CI 0.98–1.17, *p* = 0.15) (Table 2). When CD and UC were examined separately, we found no significant association between DD and UC (OR 1.10, 95% CI 0.94–1.20, *p* = 0.36) or between DD and CD (OR 1.03, 95% CI 0.92–1.16, *p* = 0.62) (Table 2). Cochran’s Q test showed no heterogeneity, and the Egger intercept method was used to assess horizontal pleiotropy in the MR-PRESSO model. The results indicated that the instrumental variables did not significantly influence the outcome through pathways other than exposure (Table 2; Figure 2).

**TABLE 2:** MR estimates of assessing the causal effect of DD on IBD.

Outcome	Method	OR	95% CI	*p*-Value	Cochran Q (*p*-value)	MR-Egger intercept (*p*-value)
Inflammatory bowel disease	MR Egger	1.15	0.90–1.49	0.27		0.51
	Inverse variance weighted	1.06	0.98–1.17	0.15	0.81	
	Maximum likelihood	1.06	0.98–1.17	0.15		
	Weighted median	1.09	0.96–1.23	0.20		
	Simple median	1.05	0.93–1.18	0.42		
Ulcerative colitis	MR Egger	1.07	0.75–1.54	0.69		0.92
	Inverse variance weighted	1.10	0.94–1.20	0.36	0.14	
	Maximum likelihood	1.10	0.95–1.17	0.31		
	Weighted median	1.09	0.94–1.27	0.25		
	Simple median	1.03	0.88–1.20	0.69		
Crohn’s disease	MR Egger	1.33	0.93–1.90	0.12		0.14
	Inverse variance weighted	1.03	0.92–1.16	0.62	0.35	
	Maximum likelihood	1.03	0.92–1.16	0.60		
	Weighted median	1.05	0.89–1.24	0.57		
	Simple median	1.01	0.85–1.19	0.95		

### Effects of IBD and its subtypes on DD

Applying the same selection criteria, we first performed a linkage disequilibrium test to identify SNPs that are associated with both IBD (including UC and CD) and DD. SNPs that had an F-statistic below 10 were then eliminated. According to the PhenoScanner database, no confounding factors were identified. Finally, using the MR-PRESSO outlier test, we identified an outlier (rs281379) associated with CD. Ultimately, we obtained 56, 35, and 49 SNPs associated with IBD, UC, and CD with DD, respectively (shown in Supplementary Table S2). When using DD as the outcome, we did not find any causal link between genetically determined IBD (including CD and UC) and DD in the outcome database when we analyzed the data, as shown in the scatter plot and forest plot (shown in Supplementary Figure S1). The Egger test indicated no evidence of potential horizontal pleiotropy in the reverse MR analysis. Cochran’s Q test and the funnel plot analysis (shown in Supplementary Figure S2) showed significant heterogeneity. Consequently, we utilized an IVW model with random effects to ascertain the magnitude of the MR effect. Our analysis did not elucidate any causal link between IBD (comprising CD and UC) and DD (Table 3). The consistent results were maintained in the leave-one-out analysis after removing individual SNPs (shown in Supplementary Figure S1).

**TABLE 3:** MR estimates of the relationship of genetically predicted IBD on DD.

Exposure	Method	OR	95% CI	*p*-Value	Cochran Q (*p*-value)	MR-Egger intercept (*p*-value)
Inflammatory bowel disease	MR Egger	1.03	0.97–1.10	0.39		0.58
	Inverse variance weighted	1.01	0.99–1.03	0.31	0.002	
	Maximum likelihood	1.01	0.99–1.03	0.19		
	Weighted median	1.01	0.99–1.04	0.29		
	Simple median	1.01	0.99–1.04	0.32		
Ulcerative colitis	MR Egger	1.05	0.97–1.14	0.18		0.17
	Inverse variance weighted	1.00	0.98–1.02	0.92	0.02	
	Maximum likelihood	1.00	0.98–1.02	0.91		
	Weighted median	0.98	0.96–1.01	0.24		
	Simple median	0.98	0.96–1.01	0.26		
Crohn’s disease	MR Egger	0.98	0.94–1.02	0.28		0.29
	Inverse variance weighted	1.00	0.98–1.02	0.80	0.02	
	Maximum likelihood	1.00	0.98–1.01	0.76		
	Weighted median	0.99	0.97–1.01	0.47		
	Simple median	1.00	0.98–1.03	0.73		

## Discussion

DD and its associated complications persistently impose a strain on the worldwide healthcare infrastructure and maintain their status as one of the most prevalent diseases in the Western hemisphere. As the eighth most common outpatient diagnosis, DD accounts for over two million annual outpatient visits in the United States (Peery et al., 2015). Despite the significant social and economic burden posed by diverticular disease, medical attention and research on its etiology and pathophysiology have been limited, leading to a dearth of information regarding its potential association with gastrointestinal risks (Wheat and Strate, 2016). As a result, there is limited information available regarding the potential association between DD and disorders of the gastrointestinal tract. While a few researchers have studied the relationship between diverticular disease and gastrointestinal conditions, their focus has primarily been on colorectal cancer and irritable bowel syndrome (IBS) (Yamada et al., 2014; Zhang et al., 2023). Additionally, previous literature has predominantly presented observational associations, limiting our understanding of the potential reverse causality in these relationships.

Based on our understanding, our research signifies the inaugural application of MR methodology for a comprehensive investigation into the causal impacts between IBD (encompassing UC and CD) and DD. When juxtaposed with preceding observational studies that probed the association between DD and IBD, the employment of the MR design diminishes the vulnerability to confounding variables and biases. Although the genetic susceptibility of IBD may have a subtle contribution to alterations in intestinal wall structure, our MR study suggests that there is likely no causal association between DD and IBD. Furthermore, we conducted sensitivity analyses to ensure the validity of our assumption.

Early reports on the potential association between DD and IBD can be traced back to the 1960s (Schmidt et al., 1968). Previous publications have indicated a 1% overlap in the diagnostic results of DD, UC, and CD (West and Losada, 2004). In recent years, several epidemiological studies have begun to examine the link between IBD and DD. A retrospective matched cohort study, involving 314 cases and 1,023 healthy controls, concluded that the incidence of DD was significantly higher in the control group compared to the IBD group (15% vs. 3.5%, *p* < 0.001) (Lahat et al., 2007). Simultaneously, conflicting results have been published regarding the incidence and clinical relevance of DD in UC patients, although most authors report a lower incidence of DD in UC patients compared to the general population. A prospective study conducted by Cassieri C between 1 January 2014, and 31 December 2014, found a significantly lower incidence of DD in UC patients compared to the control group (27.8% vs. 10.8%, *p* < 0.0001) (Cassieri et al., 2016). Another prospective study by Rispo A also demonstrated a significantly lower incidence of DD in UC patients compared to non-UC patients (8.2% vs. 28.2%; *p* < 0.001; RR, 3.4; 95CI, 1.56–7.52) (Rispo et al., 2007). Researchers, making use of cross-sectional observational or case-control study designs, have uncovered a negative correlation between DD and IBD. This suggests that IBD might not contribute as a risk factor for the development of DD; on the contrary, it could potentially serve as a protective factor. In contrast, Kinnucan and others conducted a retrospective analysis of patients who underwent colonoscopy between January 2006 and December 2013. They found that, compared to non-IBD patients, although the proportion of DD was higher, after adjusting for age and gender, the relative risk (RR) of DD in UC patients was 1.02 (95% CI, 1.02–1.03), with no significant difference in disease duration and extent between the two groups (Kinnucan et al., 2015). These conflicting findings may be attributed to the inherent limitations of the study design, such as residual confounding factors. Although these studies attempted to match for age and ethnicity to minimize confounding effects. Additionally, in 2018, Nisene and others conducted an observational case-control study to assess the incidence, characteristics, and risk factors of DD in UC patients, comparing them to a healthy control group who underwent colonoscopy. The authors found a lower incidence of DD in UC patients compared to the control group, matched for age and gender (Nascimbeni et al., 2018). However, some studies suggest that DD is more common in patients with CD, and it is difficult to differentiate between active CD and diverticulitis even in the absence of symptomatic diverticula, although there is currently no research revealing the associated incidence (Peppercorn, 2004).

Despite a considerable number of observational studies, no causal relationship between DD and IBD (including UC and CD) has been observed thus far. Several potential mechanisms can be proposed to explain the association between DD and IBD. One study explored the potential interaction between IBD and DD in terms of inflammation development and nature. It suggests that the combination of higher colonic intraluminal pressure (due to hard stools) and thinner intestinal wall (less resistance to pressure) which gives rise to diverticula may be altered in IBD patients. It is possible that loose stool consistency in IBD patients leads to lower colonic intraluminal pressure, and the chronic inflammation results in thicker intestinal walls, providing stronger resistance to luminal pressure (Lahat et al., 2007). Thus, it is hypothesized that IBD patients may have a lower risk of developing DD. However, the attention of researchers has been drawn to a case report describing a patient who previously had complicated diverticulitis requiring surgical management but later developed UC and subsequently developed asymptomatic distal colitis, although there is currently no evidence indicating that DD leads to the occurrence of IBD (Hasan and Ur Rahman, 2022). Unfortunately, there is also a lack of research on the incidence of IBD in patients with DD.

However, most previous studies have been observational, and the causal relationship remains uncertain. Contrary to previous hypotheses, our MR analysis does not support a causal relationship between IBD and DD. One of the advantages of MR analysis is that genetic variations associated with the risk factor are randomly assigned at birth, making the impact of the risk factor lifelong. This reduces the influence of confounding factors and reverse causality, providing more reliable evidence compared to traditional observational studies. Within the MR methods, the IVW method usually boasts superior statistical power compared to other MR approaches, especially the MR-Egger method (Lin et al., 2021). Hence, the IVW method is typically employed as the principal method to pinpoint potentially significant discoveries. We also conducted other MR methods and sensitivity analyses to ensure the robustness of the IVW estimates. Our study also adhered to the consistent beta direction requirement, as is the case in most MR analyses (Chen et al., 2022b; Venkatesh et al., 2022). Additionally, the large sample size is another strength of this study, which reduces sampling errors. Further research is needed to elucidate the driving factors that may have led to bias or confounding in previous observational studies.

There could be several explanations for the discrepancy between our findings and the hypotheses. Firstly, observational studies cannot eliminate the influence of certain confounding factors. Presently, it is posited that the incidence and progression of diverticulosis bear a connection to dietary constituents, contributing to alterations in colonic motility and eventually culminating in structural modifications of the colonic wall (Barbaro et al., 2022). Unfortunately, previous studies did not report the dietary information of the patients, nor did they adjust for the severity of inflammation over time or consider other factors that may affect the incidence of DD, such as genetics or exposure to nonsteroidal anti-inflammatory drugs. Considering that these factors could impact researchers’ understanding of the causes of DD. Secondarily, the symptomatic indications of IBD could effortlessly be conflated with DD or diverticulitis, culminating in the formation of an “overlap hypothesis” intertwining these medical conditions and segmental colitis associated with diverticula (SCAD) (Commane et al., 2009). The concept of inflammation within diverticular segments has been established, referred to as diverticular (disease-associated) colitis or diverticulitis-associated segmental colitis (Goldstein et al., 2000; West and Losada, 2004). It often resembles UC or CD macroscopically and microscopically, thus being misreported as diverticular UC or CD. Therefore, it is important to better understand the coexistence of these conditions and recognize common risk factors or distinguishing features (Chiba et al., 2010). Lastly, there are evident differences in the pathogenesis of IBD and DD. As mentioned earlier, the etiology of DD may be multifactorial, and the mechanisms behind the development of different disease presentations may also vary. Studies have indicated that alterations in connective tissue abnormalities, such as changes in the cross-linking of elastin (a crucial extracellular matrix protein that provides resilience and elasticity to tissues and organs), may predispose individuals to asymptomatic diverticulosis (Wess et al., 1996). Additional changes or triggering factors, such as dysbiosis of the intestinal microbiota or drug use, may be necessary for the manifestation of symptoms or complications like acute diverticulitis and diverticular bleeding. On the other hand, the pathogenesis of IBD involves a complex interplay between genetic susceptibility and environmental influences on the microbiota, resulting in inappropriate activation of intestinal immunity through the weakening of the intestinal barrier. This generally manifests as a chronic, relapsing autoimmune disease (Ramos and Papadakis, 2019). The intricate interaction of environmental factors and genetic susceptibility in the pathogenesis of IBD and DD is not yet clearly understood. It is worth noting that the incidence of both clinical entities is increasing among young populations in industrialized countries, which suggests the possible presence of shared environmental factors in their pathogenesis. Factors such as reduced dietary fiber intake, processed foods, disruption of mucosal integrity, and exposure to antibiotics during the course of complicated diverticulitis treatment can contribute to dysbiosis and intestinal inflammation (Mutlu, 2021). In the future, it is hoped that more evidence will guide research and enhance our understanding of the underlying biological mechanisms (Tursi et al., 2020).

Our investigation employed genetic information from two research groups of European descent, thereby guaranteeing genetic uniformity and the solidity of the conclusions. On the other hand, this also restricts the applicability of the results to diverse populations. The assumed causal association between DD and IBD prevalence could manifest differently across diverse demographics. Nevertheless, due to an absence of relevant data, our study could not extend its scope to validate these findings within other population groups. It is also worth highlighting that the SNPs linked with IBD outcomes were identified based on the diagnosis and existence of IBD, as opposed to the progression or severity of the disease. Consequently, the impact of DD on the progression of IBD (which may result in increased medication, disease recurrence, or surgery) is still uncertain. As such, the findings from our study cannot be utilized to determine if IBD patients need active treatment of DD to hinder disease progression. Similarly, the reverse causality results suggest that IBD is not a risk factor for DD occurrence, but the possibility of IBD impacting the progression of DD cannot be completely ruled out. Lastly, although we matched all selected SNPs with the pheWAS database to mitigate potential confounding factors and horizontal pleiotropy, the exact biological functions of many genetic variants remain unknown, and therefore, this measure does not completely eliminate the influence of horizontal pleiotropy (Meisinger and Freuer, 2022). Furthermore, negative results from MR studies cannot completely exclude causality, as genetically driven exposure is not equivalent to exposure, and negative results are often due to the strict selection of IVs.

In conclusion, our data indicate the absence of substantial causal effects in the bidirectional association between DD and IBD, both in terms of the diseases themselves and their subtypes. DD associated with IBD may be a distinct clinical entity rather than a complication of underlying inflammatory diseases. The incidental influence of DD on IBD may be mediated by other factors, while also not excluding the possibility that both diseases act as protective factors for each other. This conclusion may have clinical significance when considering treatment approaches, particularly surgical interventions, for patients with concurrent DD and IBD.

## Data Availability

The datasets presented in this study can be found in online repositories. The names of the repository/repositories and accession number(s) can be found in the article/Supplementary Material.
