# Supplementary figures and images for: No bidirectional relationship between inflammatory bowel disease and diverticular disease: a genetic correlation and Mendelian randomization study
Source: Front Genet. 2024 Feb 14;15:1334473. doi: 10.3389/fgene.2024.1334473 (PMC10899511; doi:10.3389/fgene.2024.1334473)

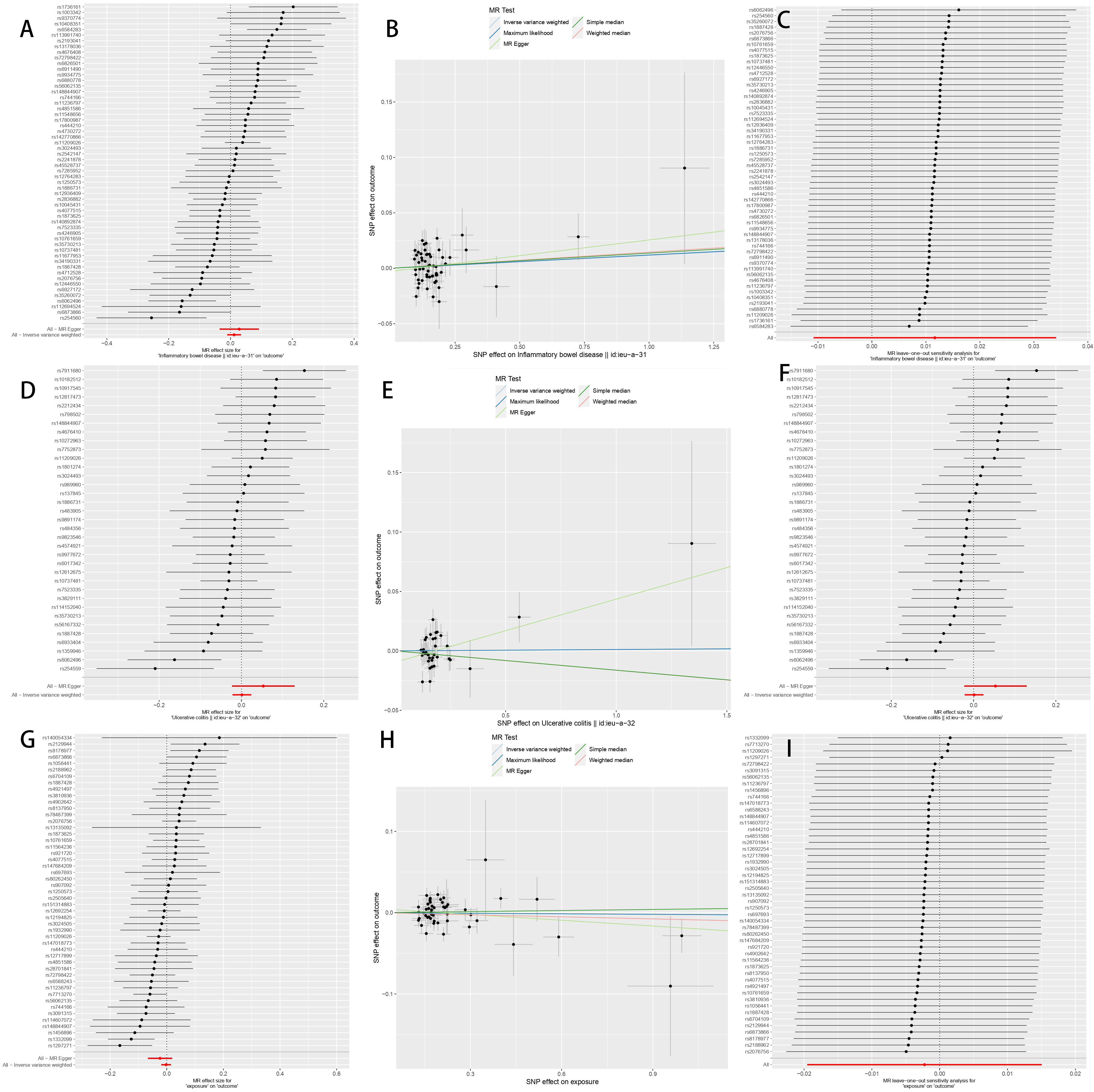

Supplement: Supplementary file 3 [file Image1.tiff]

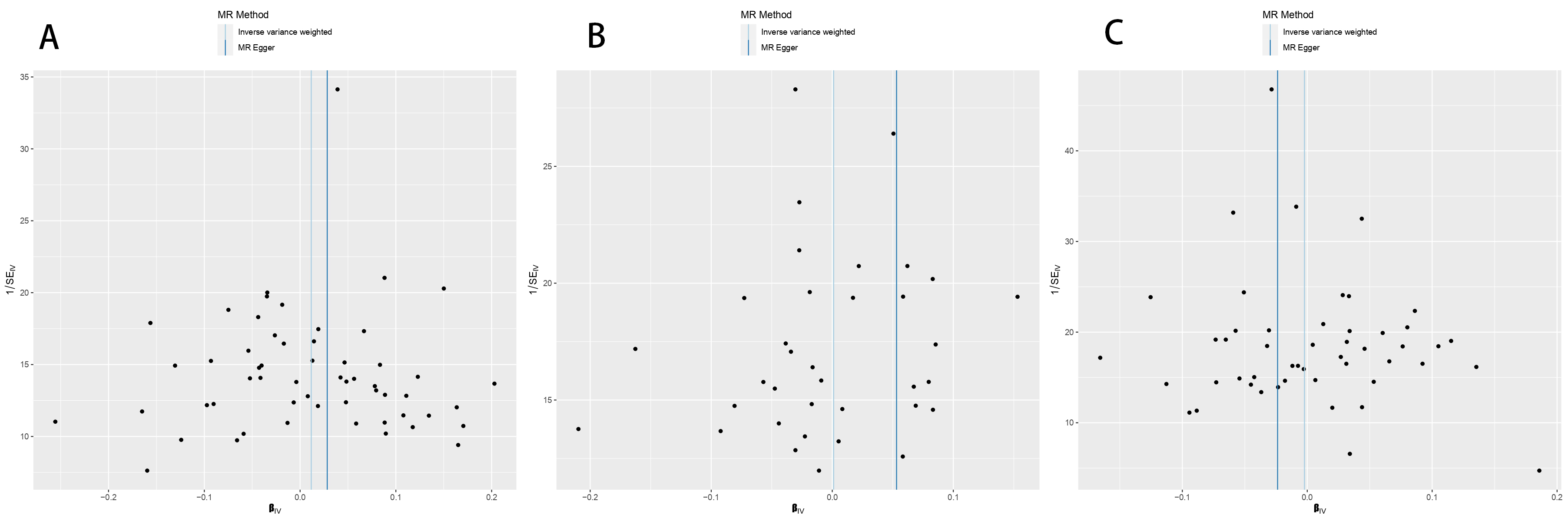

Supplement: Supplementary file 4 [file Image2.tiff]
